# Имеглимин: особенности механизма действия и потенциальные преимущества

**DOI:** 10.14341/probl12868

**Published:** 2022-03-11

**Authors:** К. О. Кузнецов, А. А. Саетова, Э. И. Махмутова, А. Г. Бобрик, Д. В. Бобрик, И. Р. Нагаев, А. Д. Хамитова, А. М. Арапиева

**Affiliations:** Российский национальный исследовательский медицинский университет им. Н.И. Пирогова; Башкирский государственный медицинский университет; Башкирский государственный медицинский университет; Башкирский государственный медицинский университет; Башкирский государственный медицинский университет; Башкирский государственный медицинский университет; Башкирский государственный медицинский университет; Башкирский государственный медицинский университет

**Keywords:** имеглимин, механизм действия, сахарный диабет 2-го типа, противодиабетическое средство, эндокринология

## Abstract

Имеглимин является первым препаратом в новом классе тетрагидротриазинсодержащих пероральных сахароснижающих средств, называемых «глиминами». Его механизм действия направлен на достижение двойного эффекта: во-первых, на улучшение функции β-клеток поджелудочной железы, во-вторых, на усиление действия инсулина в ключевых тканях, включая печень и скелетные мышцы. На клеточном уровне имеглимин модулирует митохондриальную функцию, что приводит к улучшению клеточного энергетического метаболизма, а также к защите клеток от гибели в условиях избыточного накопления активных форм кислорода. Важно отметить, что механизм действия имеглимина отличается от существующих препаратов, применяемых для лечения сахарного диабета 2-го типа. Как и препараты инкретинового ряда, имеглимин усиливает секрецию инсулина исключительно глюкозозависимым образом, однако их механизмы действия на клеточном уровне расходятся. Препараты сульфонилмочевины и глиниды функционируют путем закрытия АТФ-чувствительных калиевых каналов для высвобождения инсулина, что также отличается от имеглимина. По сравнению с метформином эффект имеглимина также значительно отличается. Другие основные классы пероральных сахароснижающих средств, такие как ингибиторы натрий-глюкозного транспортера-2, тиазолидиндионы и ингибиторы α-глюкозидазы, опосредуют свое действие через механизмы, которые не пересекаются с имеглимином. Учитывая такие различия в механизмах действия, имеглимин может быть использован в составе комбинированной терапии, например с ситаглиптином и метформином. Молекула имеглимина хорошо всасывается (Tmax-4), а период полувыведения составляет 5–6 ч, в значительной степени выводится через почки, а также не имеет клинически значимых взаимодействий ни с метформином, ни с ситаглиптином.

## Abstract (en)

Imeglimin is the first drug in a new class of tetrahydrotriazine-containing oral hypoglycemic agents called «glimines». Its mechanism of action is aimed at achieving a double effect, firstly, to improve the function of beta cells of the pancreas, and secondly, to enhance the action of insulin in key tissues, including the liver and skeletal muscles. At the cellular level, imeglimin modulates mitochondrial function, which leads to an improvement in cellular energy metabolism, as well as to the protection of cells from death in conditions of excessive accumulation of reactive oxygen species. It is important to note that the mechanism of action of imeglimin differs from existing drugs used for the treatment of type 2 diabetes mellitus. Like glucagon-like peptide-1 receptor agonists, imeglimin enhances insulin secretion in an exclusively glucose-dependent manner, but their mechanism of action at the cellular level diverges. Sulfonylureas and glinides function by closing ATP-sensitive potassium channels to release insulin, which is also different from imeglimin. Compared with metformin, the effect of imeglimine is also significantly different. Other major classes of oral antihypertensive agents, such as sodium-glucose transporter-2 inhibitors, thiazolidinediones and α glucosidase inhibitors mediate their action through mechanisms that do not overlap with imeglimine. Given such differences in the mechanisms of action, imeglimin can be used as part of combination therapy, for example with sitagliptin and metformin. The imeglimine molecule is well absorbed (Tmax-4), and the half-life is 5–6 hours, is largely excreted through the kidneys, and also has no clinically significant interactions with either metformin or sitagliptin.

## ВВЕДЕНИЕ

Имеглимин является первым препаратом в новом классе тетрагидротриазинсодержащих пероральных сахароснижающих средств, называемых «глиминами» [1]. В Японии недавно завершилась III фаза клинических исследований имеглимина, по результатам которых он показал значительную и стойкую антигипергликемическую активность у пациентов с сахарным диабетом 2-го типа (СД2), а также безопасность, хорошую переносимость и отсутствие тяжелой гипогликемии в многочисленных испытаниях, включая комбинации с метформином, ингибиторами дипептидилпептидазы-4, инсулином и другими классами противодиабетических препаратов [1–3].

Механизм действия имеглимина уникален и отличается от других основных классов пероральных сахароснижающих средств. Он нацелен на достижение двойного эффекта: потенцирование действия инсулина и устранение дисфункции β-клеток поджелудочной железы [4]. Механизм действия имеглимина в целом хорошо согласуется с нынешним пониманием патофизиологии СД2. Генетическая предрасположенность, а также факторы окружающей среды, включая переедание и низкую физическую активность, являются главными предикторами заболевания [5]. Резистентность к инсулину возникает в нескольких тканях, включая скелетные мышцы и печень, где эффект инсулина на подавление выработки глюкозы ослабевает [6].

На молекулярном уровне дисфункция является характерной чертой СД2, она способствует как формированию дефектов β-клеток [7][8], так и развитию инсулинорезистентности [9][10]. Важность митохондриальной функции подчеркивается существованием редких унаследованных форм СД2, которые являются результатом мутаций в митохондриальной ДНК [11]. Митохондриальная дисфункция проявляется несколькими способами. В некоторых тканях были описаны дефицитный окислительный метаболизм и снижение генерации АТФ наряду с более специфичными дефектами, приводящими к уменьшению или неполному окислению жирных кислот, которые являются причиной накопления липидов в клетках [12][13]. Дефекты функции митохондриальной дыхательной цепи также вызывают образование активных форм кислорода (АФК) [12], что имеет явную причинную роль в патофизиологии СД2 [14]. Никотинамидадениндинуклеотид (NAD+) является важным ко-фактором, необходимым для нормальной митохондриальной функции, а также для поддержания других основных клеточных функций [15]. Важно отметить, что нарушение метаболизма NAD+ связано с развитием многих метаболических заболеваний, включая ожирение и СД2 [16]. Кроме того, было показано, что экзогенный никотинамид, предшественник НАД+, усиливает функцию островковых β-клеток, что говорит о действии имеглимина через моделирование митохондриальной функции [17].

На сегодняшний день существует несколько классов терапевтических средств для лечения СД2 [18]. Однако, несмотря на свою неоспоримую эффективность, каждый класс имеет различного рода недостатки [18][19]. Таким образом, существует постоянная потребность в поисках новых методов лечения, нацеленных на основные аспекты патогенеза и имеющих минимальное количество побочных эффектов. Уникальный механизм действия имеглимина согласуется с современными знаниями патогенеза заболевания и может быть использован в будущей парадигме лечения СД2.

Настоящий обзор литературы выполнен с целью критической оценки собранного материала. Авторами был произведен электронный поиск публикаций в базах данных PubMed, Science Direct Scopus и Web of Science. Условиями поиска было наличие слов «Imeglimin», «type 2 diabetes» и «mechanism of action» в заголовках, аннотациях и ключевых словах. Методологическую оценку исследований проводили в соответствии со стандартами PRISMA, включая оценку систематической ошибки. Авторы независимо друг от друга проанализировали статьи, релевантные условиям поиска. Разногласия между авторами относительно приемлемости разрешались путем консенсуса. В поиск включались статьи и аннотации только на английском языке. Анализу подвергали полные тексты статей и их аннотации.

Имеглимин и устранение дисфункции β-клеток поджелудочной железы

Исследования на людях

Прямые доказательства увеличения глюкозостимулированной секреции инсулина (ГССИ) были получены в исследовании трансляционной медицины, в котором выброс инсулина в ответ на гипергликемию был существенно усилен (+112%) после 7 дней терапии имеглимином по сравнению с плацебо [20]. Кроме того, имеглимин значительно снижал соотношение проинсулин/инсулин во II фазе клинических испытаний [2], что также говорит об улучшении функции β-клеток.

Исследования на животных

Хороший эффект имеглимина в улучшении функции β-клеток поджелудочной железы был описан в нескольких исследованиях. Имеглимин снижал гипергликемию как у крыс с диабетом, индуцированным стрептозотоцином (СТЗ), так в моделях крыс Гото-Какизаки (ГК), которые характеризуются первичным дефектом количества и функции β-клеток [21]. Также в этих моделях было отмечено повышение инсулинового индекса при проведении пероральных тестов на толерантность к глюкозе [21], так же, как и в модели крысы Цукера [22]. Лечение имеглимином заметно потенциировало ГССИ in vivo как у худых, так и у крыс с избыточным весом [23]; аналогичный эффект был отмечен у мышей, употреблявших пищу с высоким содержанием жиров и сахарозы [24].

Прямой эффект имеглимина на функцию β-клеток островков поджелудочной железы был показан в нескольких аспектах. Во-первых, первая фаза глюкозозависимой секреции инсулина была увеличена до 6 раз в изолированной перфузированной модели поджелудочной железы, полученной от крыс с СТЗ-диабетом [21]. Во-вторых, прямой эффект на усиление секреции инсулина в присутствии высокого уровня глюкозы наблюдался в островках поджелудочной железы, полученных от нормальных крыс [23]. В-третьих, было установлено, что инкубация изолированных островков поджелудочной железы, полученных от крыс ГК и от крыс с СТЗ-диабетом, с имеглимином in vitro может частично восстанавливать глюкозочувствительную секрецию инсулина [25]. Необходимо отметить, что не было обнаружено никакого влияния имеглимина на секрецию инсулина при низком уровне глюкозы в отличие от препаратов сульфонилмочевины (толбутамид), которые стимулируют высвобождение инсулина из островков в модели крыс ГК, инкубированных с низким содержанием глюкозы в параллельном эксперименте.

Таким образом, многочисленные доклинические эксперименты, включающие подходы in vivo и in vitro, выявили последовательный и сильный эффект имеглимина на улучшение функции β-клеток поджелудочной железы путем усиления высвобождения инсулина исключительно глюкозозависимым способом.

Повышение количества β-клеток островков поджелудочной железы

В дополнение к прямому эффекту усиления ГССИ, который был обнаружен в вышеописанных исследованиях in vitro, был выявлен защитный эффект имеглимина, направленный на снижение гибели β-клеток в ответ на воздействие цитокинов или высокого уровня глюкозы [21]. Наличие таких эффектов свидетельствует о том, что имеглимин может иметь более долгосрочные преимущества для предотвращения потери функциональной β-клеточной массы при СД2. Чтобы подтвердить данную гипотезу, долгосрочные эффекты имеглимина были изучены в модели крыс Цукера, которая является экстремальной моделью СД2, вызванной ожирением, связанным с недостаточным количеством и повышенной гибелью β-клеток [26]. В этом контексте лечение имеглимином в течение 5 нед ослабляло снижение пула β-клеток, которое обычно наблюдается в этой модели; такой эффект, по-видимому, был вызван умеренным увеличением пролиферации β-клеток и снижением их гибели от апоптоза [22].

Имеглимин и усиление действия инсулина

Клинические данные

В III фазе клинических исследований монотерапии имеглимином в течение 24 нед было выявлено значительное влияние на индекс QUICKI (Quantitative insulin sensitivity check index), которое коррелировало с результатами глюкозного клэмп-теста [27]. В частности, средние значения QUICKI были увеличены на 0,0093 у пациентов, получавших имеглимин, по сравнению с пациентами, получавшими плацебо (P=0,005) спустя 24 нед терапии [28]. Аналогичное влияние на индекс Stumvoll — альтернативная расчетная оценка чувствительности к инсулину [29] — было отмечено во II фазе клинических исследований [28].

Функциональные эффекты в животных и клеточных моделях

В дополнение к заметному эффекту устранения дисфункции β-клеток имеются данные, которые указывают на то, что имеглимин может усиливать действие инсулина.

В модели мышей, употреблявших пищу с повышенным содержанием жиров и сахарозы, лечение имеглимином усиливало сахароснижающий эффект экзогенного инсулина [24]. Также сообщалось об инсулиносенсибилизирующем эффекте молекул в печени и скелетных мышцах; это было определено путем измерения степени фосфорилирования протеинкиназы B в ответ на экзогенный инсулин [24]. В той же модели длительное лечение имеглимином уменьшало стеатоз печени, что свидетельствует об улучшении чувствительности печени к инсулину [24]. На фоне лечения имеглимином в течение 45 дней было отмечено повышение поглощения 14С-2-дезоксиглюкозы скелетными мышцами in vivo у крыс с СТЗ-диабетом [21]; этот эффект согласуется с улучшением чувствительности к инсулину, но также, вероятно, на него повлияло улучшение секреции инсулина. Также было показано, что имеглимин опосредует инсулиноподобный эффект на поглощение глюкозы in vitro [21].

Имеглимин оказывает дозозависимое ингибирование синтеза глюкозы в печени, что было доказано на гепатоцитах, полученных от первично культивируемых крыс (Wistar); аналогичный эффект был показан при инкубации in vitro срезов печени, полученных от инсулинорезистентных крыс Цукера [21]. Вышеупомянутый эффект имеглимина на ингибирование глюконеогенеза в изолированных гепатоцитах крыс также был воспроизведен в других экспериментах [30] и, по-видимому, аналогичен эффекту метформина; однако основные механизмы действия имеглимина и метформина различны (табл. 1).

**Table:** Таблица 1. Сравнительная характеристика механизма действия имеглимина и метформина

Имеглимин	Метформин
In vivo (клинические исследования)
↑ Глюкозозависимая секреция инсулина [20].	Не сообщалось о влиянии на секрецию инсулина [20][52].
Доказано снижение инсулинорезистентности — QUICKI, Stumvoll [28]	Нет явного увеличения чувствительности к инсулину [52]
In vivo (доклинические исследования)
↑Глюкозозависимая секреция инсулина [24].	Не влияет на секрецию инсулина [52]
↑Утилизация глюкозы.↑Чувствительность к инсулину. ↑Передача сигналов инсулина [21][24]	± Повышение чувствительности к инсулину [52]
Влияние на клетки и органы
↑Глюкозозависимая секреция инсулина (островки/перфузия поджелудочной железы) [21][23][25].	Не влияет на глюкозозависимую секрецию инсулина [52].
Протекция островковых β-клеток; сохранение пула β-клеток [21][22].	Защита β-клеток in vitro [53][54]; неизвестно влияние in vivo на пул β-клеток [52].
↑Поглощение глюкозы мышцами [21].	± ↑Поглощение глюкозы мышцами [52].
↓Глюконеогенез (гепатоциты) [21]	↓Глюконеогенез (гепатоциты) [52]
Внутриклеточное действие
Конкурентное/частичное ингибирование митохондриального комплекса I; отсутствие снижения митохондриального дыхания; снижение образования АФК [24][28][32][33].	Неконкурентное ингибирование митохондриального комплекса I; снижение митохондриального дыхания [34][52]; снижение образования АФК [55].
Не влияет на митохондриальный глицерофосфат [28].	Протекция β-клеток in vitro [53][54]; неизвестно влияние in vivo на пул β-клеток [52].
Увеличение синтеза NAD+; повышение внутриклеточной концентрации Ca++ [22]	Не увеличивает концентрацию внутриклеточного Ca++ [56], не влияет на синтез NAD+ [57]

В другом экспериментальном исследовании проводили эугликемический гиперинсулинемический клэмп для оценки влияния терапии имеглимином на чувствительность к инсулину. После 2 нед лечения крыс с СТЗ-диабетом общая скорость инфузии глюкозы, необходимая для поддержания эугликемии, была значительно увеличена (+215%; P<0,01), что указывает на существенное улучшение чувствительности всего организма к инсулину. Базальная эндогенная продукция глюкозы существенно не пострадала; однако при наличии гиперинсулинемии у крыс, получавших имеглимин, было отмечено значительное снижение производства глюкозы в печени по сравнению с контрольной группой (−40%; P<0,05) [28]. Напротив, R.J. Perry и соавт. не наблюдали значительного влияния имеглимина на весь организм или на чувствительность печени к инсулину у крыс c избыточным весом после 2 нед терапии [23]. Причины такого несоответствия неизвестны, но они, вероятно, обусловлены различными видами исследуемых крыс, разными путями введения препарата, а также клэмпом на разных уровнях глюкозы. Кроме того, могут потребоваться более длительные периоды лечения для получения четкого воздействия на чувствительность к инсулину, поскольку улучшение у мышей в исследовании G. Vial и соавт. наблюдалось после 6 нед лечения имеглимином [24].

В целом имеющиеся данные свидетельствуют о том, что имеглимин усиливает действие инсулина in vivo. Также были описаны прямые эффекты подавления глюконеогенеза в гепатоцитах и стимулирования поглощения глюкозы в клетках скелетных мышц.

Молекулярные механизмы действия имеглимина

Улучшение функции митохондрий

Учитывая влияние имеглимина на разные органы и типы клеток, неудивительно, что эффекты, связанные с митохондриальной дисфункцией, которая выявляется в нескольких тканях при СД2 [30], могут лежать в основе положительных плейотропных фенотипических изменений, возникающих при лечении имеглимином. На рис. 1 представлены молекулярные механизмы действия имеглимина, приводящие к модуляции митохондриальной функции.

**Figure:**
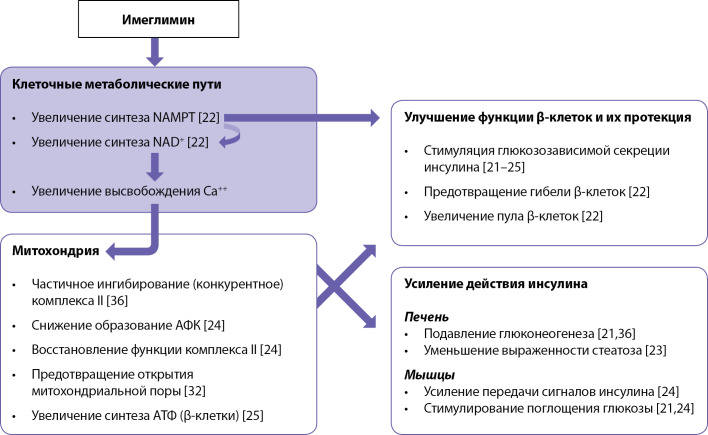
Рисунок 1. Молекулярные механизмы действия имеглимина.

В пораженных островках поджелудочной железы имеглимин усиливает генерацию АТФ и увеличивает соотношение АТФ/АДФ, что приводит к улучшению функции митохондрий [25]. Имеглимин также способствовал синтезу NAD в островках поджелудочной железы крыс ГК [25]. Содержание NADH в клетках не изменялось, однако соотношение NAD/NADH было увеличено на 30% [25]. Несмотря на отсутствие увеличения чистого количества NADH, расширение пула NAD предполагает возможность увеличения транспорта восстановительных эквивалентов, необходимых для стимулирования окислительного фосфорилирования через дыхательную цепь переноса электронов [31].

G. Vial и соавт. исследовали изменение клеточного дыхания с использованием митохондрий, выделенных из печеночной ткани мышей с СД2, на фоне лечения имеглимином [24]. Авторы выявили, что имеглимин восстанавливал дефицитную активность комплекса III при частичном ингибировании комплекса I. Такой эффект ребалансировки был связан со значительным снижением избыточного образования АФК. Также было отмечено положительное влияние имеглимина на компоненты митохондриальной структуры (например, содержание кардиолипина) [24]. В эксперименте с использованием эндотелиальных клеток человека (HMEC-1) эффект имеглимина на подавление образования АФК путем уменьшения обратного переноса электронов через комплекс I был реплицирован, сокращения потребления клеточного кислорода не отмечалось [32].

Известно, что повышенная выработка АФК улучшает открытие митохондриальной поры (МП). Это происходит при различных патологических состояниях и может привести к высвобождению проапоптотических белков в цитозоль, что ведет к гибели клеток [33][34]. В клетках HMEC-1 имеглимин эффективно предотвращал открытие МП, что приводило к снижению гибели клеток [32]. На основании этих данных можно предположить, что вышеупомянутое действие имеглимина, направленное на предотвращение гибели β-клеток в культивируемых островках поджелудочной железы [21], может быть обусловлено его влиянием на открытие МП, однако, поскольку это очень разные типы клеток, для подтверждения данной гипотезы потребуются дальнейшие исследования.

Наконец, эффект имеглимина, направленный на ингибирование глюконеогенеза в гепатоцитах, а также его потенциальный эффект, направленный на подавление выхода глюкозы из печени in vivo, могут быть связаны с частичным ингибированием комплекса I дыхательной цепи. Метформин также ингибирует глюконеогенез посредством блокирования комплекса I [35]. В изолированных гепатоцитах крыс как имеглимин, так и метформин ингибируют глюконеогенез, однако их влияние на комплекс I, по-видимому, расходится. Измеряя сродство NADH к дыхательной цепи в пермеабилизированных гепатоцитах, G. Vial и соавт. обнаружили, что имеглимин уменьшал сродство NADH к дыхательной цепи, но не влиял на Vmax (конкурентное ингибирование), тогда как метформин снижал как Vmax, так и аффинность (неконкурентное ингибирование) [36]. Учитывая, что имеглимин производит мягкое, конкурентное ингибирование комплекса I, не влияя на общее потребление клеточного кислорода, авторы предположили, что он вызывает кинетическое ограничение дыхательной цепи, которое не влияет на ее максимальную активность, но может быть достаточным для воздействия на глюконеогенез в гепатоцитах. Метформин, напротив, является неконкурентоспособным ингибитором дыхательной цепи и значительно снижает скорость потребления кислорода клетками [36]. Эти данные согласуются с результатами других исследований, полученными с использованием клеток HMEC-1, где метформин, но не имеглимин, вызывал умеренное снижение потребление клеточного кислорода [32].

Вышеупомянутые выводы поднимают ряд важных вопросов, на которые еще предстоит ответить в будущем. Какой молекулярный механизм/механизмы могут объяснить наблюдаемое воздействие на комплекс I (или комплекс III)? Существуют ли тканеспецифичные различия во влиянии имеглимина на митохондриальную функцию? Если уровни АТФ подавляются в гепатоцитах, то почему наблюдалось их повышение в островках поджелудочной железы? В гепатоцитах исследовали высокие концентрации (100–1000 мкМ); в островках поджелудочной железы эффекты имеглимина наблюдались при концентрации 25–100 мкМ. Кроме того, увеличение клеточного NAD до настоящего времени наблюдалось только в островках. Влияние имеглимина на повышение активности комплекса III также предполагает, что чистое воздействие на синтез АТФ может быть связано со степенью исходной дисфункции в различных компонентах дыхательной цепи (т.е. существует ли основной дисбаланс в комплексе I по сравнению с комплексом III, который может быть восстановлен до нормы). Прямую связь между влиянием имеглимина на митохондриальную функцию и усилением ГССИ или действия инсулина in vivo еще предстоит установить.

Повышение уровня лактата плазмы и даже лактат-ацидоз могут возникать при передозировке метформина или в условиях почечной недостаточности [37][38]. Поскольку ингибирование комплекса I может способствовать риску возникновения таких осложнений [38], важно учитывать, что имеглимин также может приводить к их возникновению. Интересно, что метформин также ингибирует митохондриальную глицерофосфатдегидрогеназу (мГФД), эффект, который также может приводить как к ингибированию глюконеогенеза, так и к накоплению лактата [39]. Однако было выявлено, что имеглимин не оказывал влияния на мГФД крыс по сравнению с метформином, который действовал так, как ожидалось [28].

Несмотря на наличие некоторых сходств в химических структурах, большинство эффектов имеглимина in vivo и in vitro отчетливо отличаются от метформина, основываясь на имеющихся данных литературы. Как метформин, так и имеглимин имеют общие эффекты, направленные на ингибирование комплекса I; однако конкретные механизмы расходятся. Таким образом, имеющиеся данные убедительно свидетельствуют о том, что имеглимин не провоцирует увеличение лактата и имеет более низкий риск возникновения лактат-ацидоза по сравнению с метформином.

Потенциальная роль других механизмов в улучшении функции β-клеток

Как упоминалось выше, имеглимин увеличивает клеточный пул NAD в изолированных островках, полученных от крыс ГК. В эксперименте было показано, что имеглимин увеличивает синтез NAD, индуцируя экспрессию никотинамидфосфорибозилтрансферазы (NAMPT), ключевого фермента в пути трансфосфорилирования [25]. В дополнение к своей роли в усилении митохондриальной функции [40] NAD метаболизируется CD38 [41] для генерации второго мессенджера циклической АДФ-рибозы, которая участвует в усилении мобилизации Ca из внутриклеточного пула посредством взаимодействия с рецептором рианодина [42]. Увеличение внутриклеточной концентрации Ca требуется для ГССИ [43] и потенцируется имеглимином, а также другими стимулами, включая инкретины [25]. Для дальнейшего изучения возможной связи между эффектами имеглимина, направленными на увеличение NAD, и стимулированием мобилизации Ca проводились дополнительные исследования [25]. Частичный нокдаун CD38, по-видимому, блокирует влияние имеглимина на ГССИ; избыток рианодина также использовался для блокировки рианодиновых рецепторов, что приводило к снижению способности имеглимина усиливать ГССИ. Учитывая полученные результаты, можно предположить, что влияние имеглимина на ГССИ опосредовано через этот путь [25]. Хотя увеличение синтеза NAD может способствовать усилению ГССИ, этот путь не объясняет другие эффекты имеглимина (например, сенсибилизацию инсулина в других клетках и тканях). Возможное влияние имеглимина на NAD в других тканях еще предстоит исследовать.

Важно отметить, что влияние имеглимина на ГССИ оказалось устойчивым к диазоксиду, тогда как действие препаратов сульфонилмочевины было полностью ингибировано диазоксидом [25]. Таким образом, преобладающее действие имеглимина в островках, по-видимому, не зависит от классического «запускающего» пути, включающего закрытие АТФ-чувствительных калиевых каналов [44]. Существует явное сходство между эффектом имеглимина, направленным на усиление ГССИ, и эффектами глюкагоноподобного пептида-1, который также устойчив к диазоксиду [45]; однако имеглимин не повышал цАМФ, который является облигатным медиатором действия инкретина [25].

В целом молекулярная основа эффекта имеглимина, направленного на улучшение функции β-клеток, согласуется с его способностью модулировать митохондриальную функцию, а также увеличивать мобилизацию Ca, что может быть связано с синтезом и метаболизмом NAD. Хотя можно ожидать, что увеличение митохондриального дыхания вызовет высвобождение инсулина через закрытие АТФ-чувствительного калиевого канала, хорошо известно, что дополнительные анаплеротические митохондриальные метаболические пути могут привести к независимой от АТФ-чувствительных калиевых каналов амплификации ГССИ [46].

В дополнение к потенциальному влиянию этого механизма на функцию β-клеток можно предположить, что вышеупомянутые эффекты имеглимина, направленные на протекцию β-клеток, могут быть частично обусловлены увеличением внутриклеточной концентрации NAD. Действительно, известно, что истощение NAD потенциирует апоптотическую гибель клеток [47], а экзогенный NAD обладает цитопротекторными свойствами [48].

Дополнительные эффекты и потенциальные преимущества имеглимина

В свете доказательств, отражающих то, что имеглимин модулирует митохондриальную функцию, снижает АФК и обладает цитопротекторными свойствами, его потенциал протекторного действия может распространяться и на другие ткани, которые поражаются при СД2. Используя крыс Цукера, M. Lachaux и соавт. описали положительный эффект при сердечной дисфункции на фоне лечения имеглимином [49]. Специфические улучшения включали снижение конечного диастолического давления в левом желудочке (ЛЖ) и увеличение перфузии миокарда ЛЖ; кроме того, параллельно наблюдалось снижение АФК. В том же эксперименте было показано, что имеглимин увеличивает эндотелий-зависимую релаксацию коронарных артерий. Важно отметить, что после 90 дней лечения имеглимином в данной модели частично нормализовалась гиперальбуминурия, со среднего значения 385 до 251 мг/сут (P<0,05); среднее значение у контрольных крыс составляло 108 мг/сут. Эта очевидная польза для почек также была связана со значительным уменьшением интерстициального фиброза почек, однако не наблюдалось улучшения канальцевых повреждений и интерстициального воспаления [49]. Таким образом, имеглимин может обладать дополнительным потенциалом для лечения важных осложнений сахарного диабета, включая сердечную дисфункцию и нефропатию. Имеглимин обладает хорошей безопасностью для сердечно-сосудистой системы, на сегодняшний день не наблюдалось каких-либо побочных эффектов, и последние клинические данные также указывают на отсутствие удлинения интервала Q–T, а также других аномалий на ЭКГ при лечении имеглимином [50].

В табл. 2 представлена сравнительная характеристика основных групп пероральных сахароснижающих препаратов.

**Table:** Таблица 2. Сравнительная характеристика основных групп пероральных сахароснижающих препаратов

Терапевтический класс	Клинический эффект снижения уровня HbA1c, %	Побочные эффекты	Механизм (ы) действия
Бигуаниды (метформин)	от −0,7 до 1,2% [58]	Возможный риск лактат-ацидоза; побочные эффекты со стороны желудочно-кишечного тракта [58]	См. табл. 1
Препараты сульфонилмочевины, глиниды	≈−1,0 с потенциальной потерей эффекта с течением времени [59]	Гипогликемия; увеличение веса; повышенный риск сердечно-сосудистой смертности [59]	Связывание субъединицы K+-ТФ → закрытие канала → глюкозонезависимая секреция инсулина [44]
Ингибиторы натрий-глюкозного котранспортера-2	от −0,6 до 0,9% [60]	Положительное влияние на почки и сердечно-сосудистую систему; легкая потеря веса; снижение гликемической эффективности при почечной недостаточности	Ингибирует реабсорбцию глюкозы в почках; повышение чувствительности к инсулину [61]
Ингибиторы α-глюкозидазы	от −0,44 до 1,0% (−0,78% при дозе 100 мг три раза в день) [62]	Побочные эффекты со стороны желудочно-кишечного тракта (до 74%); небольшая потеря веса [62]	Ингибирует переваривание углеводов в кишечнике [63]
Агонисты рецепторов ГПП-1	от −0,8 до 1,4% [64]	Потеря веса; снижение сердечно-сосудистого риска [64]	Передача сигналов цАМФ → увеличение ГССИ [64]
Ингибиторы ДПП4	от −0,6 до 0,8% [65]	Потенциальная потеря эффективности через 9–12 мес	Стабилизация и повышение концентрации инкретина, что приводит к ↑ ГССИ [66]
Тиазолидиндионы	от −1,0 до 1,6% [67]	Увеличение веса; отек; повышенный риск переломов костей	Агонисты PPARγ → повышение сенсибилизации к инсулину [68]
Имеглимин	от −0,94 до 1,0% [3]	На сегодняшний день сообщения о побочных эффектах имеглимина отсутствуют	Модуляция митохондриальной функции; увеличение синтеза АТФ и НАД+ (в островках) → увеличение ГССИ; усиление действия инсулина

## ЗАКЛЮЧЕНИЕ

Имеглимин является первым в своем классе пероральным препаратом, нацеленным сразу на несколько ключевых компонентов патофизиологии СД2. Его механизм действия направлен на достижение двойного эффекта, во-первых, на улучшение функции β-клеток поджелудочной железы, во-вторых, на усиление действия инсулина в ключевых тканях, включая печень и скелетные мышцы. На клеточном уровне имеглимин модулирует митохондриальную функцию, что приводит к улучшению клеточного энергетического метаболизма, а также к защите клеток от гибели в условиях избыточного накопления АФК.

Важно отметить, что механизм действия имеглимина отличается от существующих препаратов, применяемых для лечения СД2. Как и препараты инкретинового ряда, имеглимин усиливает секрецию инсулина исключительно глюкозозависимым образом, однако, их механизм действия на клеточном уровне расходится. Препараты сульфонилмочевины и глиниды функционируют путем закрытия АТФ-чувствительных калиевых каналов для высвобождения инсулина, что также отличается от имеглимина. По сравнению с метформином эффект имеглимина также значительно отличается. Другие основные классы пероральных сахароснижающих средств, такие как ингибиторы натрий-глюкозного транспортера-2, тиазолидиндионы и ингибиторы α-глюкозидазы, опосредуют свое действие через механизмы, которые не пересекаются с имеглимином. Учитывая такие различия в механизмах действия, имеглимин может быть использован в составе комбинированной терапии, например с ситаглиптином и метформином. Молекула имеглимина хорошо всасывается (Tmax-4), а период полувыведения составляет 5–6 ч, в значительной степени выводится через почки, а также не имеет клинически значимых взаимодействий ни с метформином, ни с ситаглиптином [51].

Учитывая профиль переносимости и последовательную эффективность снижения уровня глюкозы в ряде клинических испытаний, имеглимин может применяться у пациентов с резистентностью к лечению, включая пожилых людей и пациентов с почечной недостаточностью. Имеются случаи, при которых применение имеглимина может быть нецелесообразно, например, при хорошем контроле заболевания другими препаратами, а также при необходимости получения дополнительных эффектов (например, снижение веса), которые могут быть обеспечены другими классами пероральных сахароснижающих средств. У некоторых пациентов применение имеглимина может вызвать развитие побочных эффектов со стороны желудочно-кишечного тракта (диарея), хотя частота возникновения таких эффектов значительно ниже, чем при применении метформина [1]. Кроме того, на сегодняшний день не имеется данных по применению имеглимина у отдельных групп пациентов, включая детей и пациентов с печеночной недостаточностью.

Уникальный механизм действия имеглимина согласуется с существующими клиническими данными и имеет несколько важных особенностей: отсутствие явных рисков тяжелой гипогликемии и лактат-ацидоза; отсутствие побочных эффектов, связанных с механизмом действия; кардио- и нефропротекторное действие.

## ДОПОЛНИТЕЛЬНАЯ ИНФОРМАЦИЯ

Источники финансирования. Работа выполнена по инициативе авторов без привлечения финансирования.

Конфликт интересов. Авторы декларируют отсутствие явных и  потенциальных конфликтов интересов, связанных с содержанием настоящей статьи.

Участие авторов. Кузнецов К.О. — разработка концепции и дизайна исследования, получение и анализ данных, интерпретация результатов; Саетова А.А. — разработка дизайна исследования, написание статьи; Махмутова Э.И. — анализ данных, написание статьи; Бобрик А.Г. — интерпретация результатов, написание статьи; Бобрик Д.В. — получение и анализ данных, редактирование статьи; Нагаев И.Р. — интерпретация результатов, редактирование статьи; Хамитова А.Д. — анализ данных, редактирование статьи; Арапиева А.М. — получение данных, редактирование статьи. Все авторы одобрили финальную версию статьи перед публикацией, выразили согласие нести ответственность за все аспекты работы, подразумевающую надлежащее изучение и решение вопросов, связанных с точностью или добросовестностью любой части работы. Все авторы внесли равный вклад в написание статьи и одобрили ее финальную версию перед публикацией.

Благодарности. Авторы признательны д.м.н., профессору Д.А. Еникееву за существенный вклад в концепцию исследования, а также за  одобрение финальной версии рукописи.
